# Follicular dendritic cell sarcoma of the pharyngeal region

**DOI:** 10.3892/ol.2013.1224

**Published:** 2013-03-04

**Authors:** TENGPENG HU, XINHUA WANG, CHANG YU, JIAQIN YAN, XUNDONG ZHANG, LING LI, XIN LI, LEI ZHANG, JINGJING WU, WANG MA, WENCAI LI, GUANNAN WANG, WUGAN ZHAO, XIANZHENG GAO, DANDAN ZHANG, MINGZHI ZHANG

**Affiliations:** 1 Lymphoma Diagnosis and Treatment Center, Department of Oncology, The First Affiliated Hospital of Zhengzhou University, Zhengzhou, Henan 450052, P.R. China;; 2 Department of Pathology, The First Affiliated Hospital of Zhengzhou University, Zhengzhou, Henan 450052, P.R. China

**Keywords:** follicular dendritic cell sarcoma, pharyngeal, immunohistochemistry, surgery, radiotherapy, chemotherapy

## Abstract

Follicular dendritic cell sarcoma (FDCS) is a rare neoplasm arising most commonly from follicular dendritic cells in the lymph nodes. It is exceedingly rare in extranodal sites, particularly in the pharyngeal region. The present study reports 3 cases occurring in the pharyngeal region. Case 1 had tonsil and cervical lymph node involvement, while case 3 also had tonsil involvement. Cases 1 and 3 relapsed locally at 3 and 17 months after surgery, respectively. Case 2 was diagnosed with a tumor in the parapharyngeal space and the patient succumbed to the disease 5 months after treatment with combined surgery and chemotherapy. All 3 cases were misdiagnosed initially. Pathological biopsy examination, including histopathology and immunohistochemistry, was essential for diagnosis. The data for 52 cases, including cases from the literature and the present cases, were analyzed. The results indicated that 57% (26/46) of the initial diagnoses were inaccurate, while the recurrence, metastasis and mortality rates were 40, 16 and 10%, respectively. The statistics supported the theory that FDCS of the pharyngeal region is a low-grade sarcoma. Involvement of the tonsils (52%, 27/52) and parapharyngeal space (19%, 10/52) were observed most commonly, while FDCS at various sites showed different prognoses. The various survival rates were calculated in the present study. The large tumors (≥4 cm) had a poorer prognosis than the small tumors (<4 cm; P<0.05). Among the 50 cases with available follow-up data, 46% (23/50) were treated with surgery alone, 52% (26/50) with combination therapy (surgery followed by chemotherapy and/or radiotherapy) and 2% (1/50) with surveillance. There was no statistically significant evidence (P>0.05) that combination therapy improves survival rates, compared with surgery alone.

## Introduction

Follicular dendritic cells (FDCs) are non-lymphoid, non-phagocytic antigen-presenting cells of the immune system and are able to capture, process and present antigens and immune complexes to B and T cells. FDCs belong to the dendritic cell family which may be divided into 4 categories based on immunophenotypes: interdigitating dendritic, indeterminate, Langerhans and follicular dendritic cells (1). FDCs are normally located in the germinal centers of primary and secondary follicles where they form a tight meshwork with other cells. In addition, FDCs may be located extranodally in acquired lymphoid tissue which is the foundation condition for the occurrence of extranodal FDCS (2). The possibility of a follicular dendritic cell tumor was first suggested by Lennert in 1978 (3). A primary neoplasm of FDC origin in a lymph node was first described by Monda *et al* in 1986 (4), while the name, FDCS, was first proposed by Chan *et al* in 1997 (5).

FDCS is a rare neoplasm. At present, ∼200 cases have been reported in the English-language literature. The majority of cases were observed in lymph nodes and less than one-third were extranodal (6). The pharyngeal region, along with the abdominal/pelvic cavity, was one of the preferred sites of extranodal FDCS (7). The present study reports 3 cases of pharyngeal FDCS treated at the First Affiliated Hospital of Zhengzhou University (Zhengzhou, China) over the last 10 years. With regard to the diagnosis and therapeutic modalities, 49 cases reported in the English-language literature were reviewed.

## Materials and methods

### Samples

Tissue specimens from 3 patients, who were all treated at the First Affiliated Hospital of Zhengzhou University over the last decade, were collected and fixed in 10% buffered formalin, then dehydrated, embedded in paraffin and cut into 4-*μ*m-thick sections for hematoxylin and eosin (HE) staining and light microscopy.

### Immunohistochemistry

Immunohistochemical reactions were performed on the paraffinized sections using the EnVision method. The diagnostic antibodies used included antibodies against CD21, CD23, CD35, podoplanin (D2-40), CXCL-13, S100 protein, vimentin, epithelial membrane antigen (EMA), cytokeratin (CK, AE1/AE3), CD163, leukocyte common antigen (LCA, CD45), CD1a, CD68, CD34, CD3 and CD20. Ki-67 (MIB-1) antigen was detected to show the proliferative activity of each lesion.

### In situ hybridization

*In situ* hybridization for EBV-encoded RNA (EBER) was also performed on the formalin-fixed, paraffin-embedded tissue sections and the probes used were purchased from Beijing Zhongshan Golden Bridge (Beijing, China).

### Literature review

A literature search was performed using MEDLINE on PubMed (www.ncbi.nlm.nih.gov/pubmed) with the term ‘follicular dendritic cell sarcoma’ combined with ‘extranodal’ and ‘pharyngeal’. Efforts were made to identify cases that were reported more than once in various settings and only the entry with the most up-to-date information was included in such cases. Only FDCS of the pharyngeal region were included in Table III.

### Statistical analysis

Statistical analyses were performed using the Statistical Analysis System (SAS) 9.2. Disease-free survival (DFS) and overall survival (OS) rates were analyzed using the Kaplan-Meier method. Log-rank tests were used to analyze the equality of survival function over strata. P<0.05 was considered to indicate statistically significant differences.

## Results

### Clinical data

As shown in Table I, the 3 patients were all female, with ages ranging between 36 and 64 years (median, 59 years). Of the 3 cases, 2 were tonsil lesions with case 1 being a left-tonsil lesion with left cervical lymph node involvement, while case 3 was a left-tonsil lesion. Case 2 was a mass in the parapharyngeal space. A mass was detected in the oropharynx and slight dysphagia symptoms were experienced in all 3 cases. Tumor resection was performed in all 3 cases and the symptoms were relieved. The sizes of the tumors ranged between 3.0 and 6.0 cm as measured in the longitudinal dimension, with an average of 4.5 cm. All the tumors were initially misdiagnosed as non-specific inflammation (case 1), a benign tumor (case 2) or squamous cell carcinoma (case 3).

There was only 1 patient (case 1) who remained alive in the follow-up 7 to 24 months. Case 2 succumbed to the disease 7 months after initial treatment with combined surgery and chemotherapy without recurrence. Case 3 succumbed 7 months after local recurrence despite a second resection followed by radiotherapy (200 cGy x 25 times; total 5,000 cGy). Case 1 relapsed locally in the left neck lymph nodes 6 months after surgery, then salvage therapy was performed, including 4 courses of the ‘CHOP’ adjuvant chemotherapy regimen according to the following schedule: cyclophosphamide 750 mg/m^2^ i.v (day 1), vincristine 1.4 mg/m^2^ i.v (day 1), doxorubicin 40 mg/m^2^ i.v (day 1) and prednisone 100 mg p.o. (days 1–5) and 1 course of radiotherapy with a total of 5,600 cGy (200 cGy x 28 times). Subsequently, complete remission (CR) was achieved. The longest disease-free time (DFT) among the 3 present cases was 17 months.

### Histopathology

**Figure 1:**
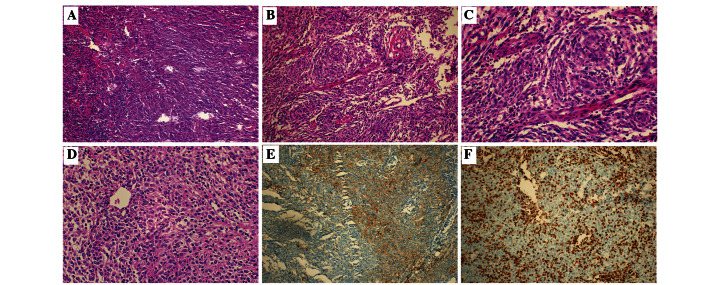
Pathological characteristics of pharyngeal FDCS. (A) Tumor cells arranged in a diffusing sheet-like distribution, partially in the storiform pattern (case 2). (B) Tumor cells arranged in the whorl pattern (case 3). (C) Indistinct cell borders and slightly eosinophil-stained cytoplasm of tumor cells. Infiltrating small lymphocytes and tissue cells were observed in the background (case 3). (D) Nuclei of tumor cells were irregular, round- or spindle-shaped, containing delicate chromatin and small nucleoli. Clear mitotic counts were observed (case 1). (E) Tumor cells were positive for podoplanin (case 3). (F) Tumor cells were negative for LCA, but the lymphocytes scattered in the background were positive for LCA (case 3). FDCS, follicular dendritic cell sarcoma; LCA, leukocyte common antigen.

In case 1, the tumor was confined by fibrous tissue and showed clear boundaries. The tumor cells were ovoid- to spindle-shaped and arranged in sheet-like or fascicular patterns. In case 2, the tumor cells showed diffusing sheet-like distributions, partially in the storiform pattern, and were separated by collagenous fibers (Fig. 1A). In case 3, whorl structures formed by tumor cells were observed (Fig. 1B). In all 3 cases, irregular round or spindle nuclei, containing delicate chromatin and small nucleoli, were observed (Fig. 1D). Indistinct cell borders and slightly eosinophil-stained cytoplasm were the most common features. Infiltrating small lymphocytes and tissue cells were distributed in the background (Fig. 1C).

### Immunohistochemistry and in situ hybridization

As shown in Table II, the immunohistochemical stains in all 3 cases were positive for CD21, CD35, CD23 and vimentin, but negative for CK (positive minority in case 3) and S100 protein. The expression of D2-40 was observed in partial or focal tumor cells in all cases while CXCL-13 was absent (Fig. 1E). Case 1 was slightly positive for EMA. CD163 and LCA were positive in the background cells(Fig. 1F). The Ki-67-labeling index (Ki-67 LI) of all the lesions ranged between 30 and 40%. Furthermore, the *in situ* hybridizations for EBER were negative in all cases. Certain antibodies were stained in 1 case but not stained in the others, as follows: in case 3, the immunohistochemical reaction for CD1a was negative, while CD3 and CD20 were positive in the background cells and negative in tumor cells; CD34 was negative in the tumor of case 2.

### Literature review of pharyngeal FDC sarcomas

The following summaries were obtained from a total of 52 cases of FDCS in the pharyngeal region, including the 3 present cases and 49 cases identified through a MEDLINE search (Table III).

**Figure 2:**
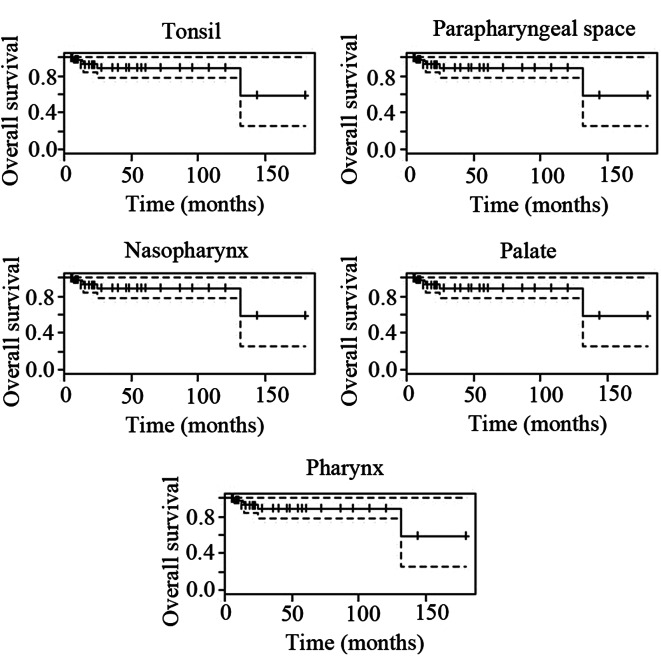
OS curves of pharyngeal FDCS at various sites. OS, overall survival; FDCS, follicular dendritic cell sarcoma.

**Figure 3:**
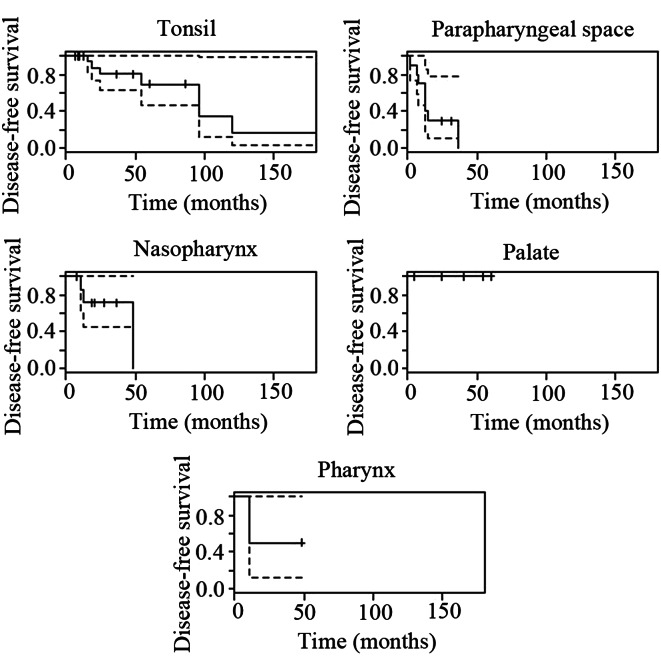
DFS curves of pharyngeal FDCS at various sites. DFS, disease-free survival; FDCS, follicular dendritic cell sarcoma.

The patients ranged between 14 and 77 years old (median, 48 years; average, 47 years). The female/male ratio was 1:1.08 (25:27). The tumors tended to occur in young to middle aged adults without any gender difference. The FDCSs occurred in various anatomical parts of the pharyngeal region. The most common site was the tonsils, with 27 cases (52%). Other sites included the parapharyngeal space (10/52, 19%), nasopharynx (8/52, 15%), palate (4/52, 8%), pharynx (2/52, 4%) and hypopharynx (1/52, 2%). The tumors in the various anatomical regions showed distinct prognoses (Table IV) and their survival curves are shown in Figs. 2 and 3. The recurrence, metastasis and mortality rates in the tonsils were 30.8, 15.4 and 3.9%, respectively. Of the cases with metastasis, all (4/4, 100%) involved the cervical lymph nodes, while a number (2/4, 50%) also involved the lungs. The FDCSs in the parapharyngeal space showed the worst prognosis with recurrence, metastasis and mortality rates of 80, 20 and 30%, respectively. Furthermore, all the cases (2/2, 100%) of metastasis from the parapharyngeal space involved the lungs. Due to the limited number of cases, the results in Table IV remain to be confirmed by a larger-scale survey.

To assess the prognosis, the size of the initial tumor was analyzed. The sizes varied between 1 and 7 cm, with an average of 3.4 cm, in the longitudinal dimension. The large tumors (≥4 cm) accounted for 34% (12/35) of cases and had recurrence, metastasis and mortality rates of 58.3, 8.3 and 33.3%, respectively. However, the small tumors (<4 cm, 66%, 23/35) exhibited recurrence, metastasis and mortality rates of 17.4, 0 and 0%, respectively. The 2-year DFS and OS rates of the large tumors were 36.8 and 59.7%, respectively, in the follow-up of 6 to 132 months, while those of the small tumors were 81.5 and 100% in the follow-up of 5 to 54 months.

**Figure 4:**
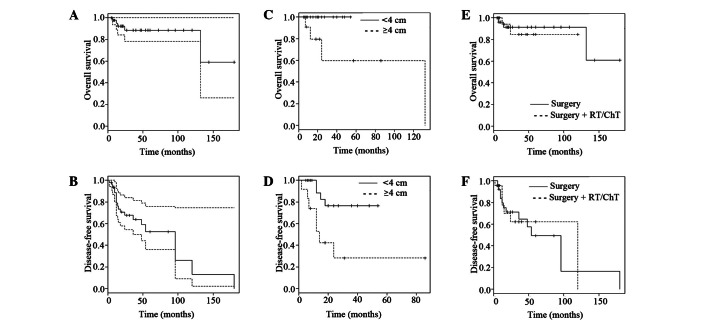
Survival curves of pharyngeal FDCS. (A) OS and (B) DFS curves of pharyngeal FDCS. (C) OS and (D) DFS curves of FDCS with various sizes (<4 cm or ≥4 cm in the longitudinal dimension). (E) OS and (F) DFS curves of FDCS with various treatments (surgery alone or surgery followed by RT and/or ChT). FDCS, follicular dendritic cell sarcoma; OS, overall survival; DFS, disease-free survival; RT, radiotherapy; ChT, chemotherapy.

Since the longest follow-up of the small tumors was 54 months (<60 months), the 5-year DFS and OS rates were not suitable, while those of the large tumors were 36.8 and 59.7%, respectively. Further statistical analysis of the size was performed among the 35 available tumors and indicated that large tumors (≥4 cm) had a poorer prognosis compared with small tumors (<4 cm) with regard to DFS (P=0.0446, P<0.05) and OS (P=0.0086, P<0.05) rates (Table IV, Fig. 4C and D; Kaplan-Meier estimation, log-rank test). Notably, 58.3% of the large FDCSs (7/12) occurred in the parapharyngeal space, among which 85.7% (6/7) exhibited recurrence. To a certain degree, this accounted for the poor prognosis of tumors in the parapharyngeal space. While 41.2% of the large sarcomas (5/12) occurred in the tonsils, only 1 case (1/5, 25%) locally relapsed.

In the 50 cases with follow-up data, the follow-up time ranged between 5 and 180 months, with a median of 23 months and an average of 39 months. A total of 20 cases (20/50, 40%) relapsed, including 12 (12/50, 24%) of local recurrence and 8 (8/50, 16%) of distant metastasis. Additionally, 5 patients (5/50, 10%) succumbed to the disease. The disease-free time varied from 2 to 180 months (median, 18 months; mean, 32 months). The recurrence, metastasis and mortality rates were 40, 16 and 10%, respectively. The 2- and 5-year DFS rates for the entire group were 66.2 and 51.3%, respectively, while the 2- and 5-year OS rates were 88.6 and 88.6%, respectively (Kaplan-Meier estimation; Table IV, Fig. 4A and B).

Initially, nearly all the cases (51/52, 98.1%) underwent surgery to remove the tumor, with the exception of 1 case (1/52, 1.9%) where the patient opted to be surveilled. A total of 2 patients were lost to follow-up. Adjuvant treatment (radiotherapy and/or chemotherapy) was administered post-operatively to over half of the cases (26/50, 52%), among which 12 (12/26, 46%) suffered recurrence (local recurrence and/or metastasis), 8 (8/26, 31%) suffered metastasis (lung, lymph nodes) and 2 (2/26, 8%) succumbed to the disease in the follow-up of 6 to 180 months. For the group who received surgery and adjuvant therapy, the recurrence, metastasis and mortality rates were 46.2, 30.8 and 7.7%, respectively. The 2- and 5-year DFS rates were 73.8 and 66.4%, respectively, while the 2- and 5-year OS rates were 91.4 and 91.4%, respectively. Among the surgery alone group, recurrence was observed in 8 cases (8/23, 35%), no cases of metastasis were detected and 2 cases (2/23, 9%) succumbed to the disease in the follow-up of 5 to 120 months. The recurrence, metastasis and mortality rates were 34.8, 0 and 8.7%, respectively. The 2- and 5-year DFS rates were 67.4 and 56.2%, respectively, while the 2- and 5-year OS rates were 84.7 and 84.7%, respectively (Table IV). The disease-free time of patients who received adjuvant treatment (range, 6 to 180 months; mean, 39 months) was longer compared with the patients who received surgery alone (range, 2 to 120 months; mean, 24 months). However, the survival curves (Fig. 4E and F) suggested that the survival rates were not significantly different (P>0.05, log-rank test).

## Discussion

FDCS of the pharyngeal region is an extremely rare neoplasm which has been detected in the tonsils, palate, parapharyngeal space, nasopharynx, pharynx and hypopharynx, with the majority of distant metastasis occurring in the cervical or axillary lymph nodes and lungs (8). Similar to other body regions, pharyngeal FDCS occurs at a wide range of ages and has an adult predominance with equal gender distribution (9). The tumor usually presents as an enlarging solid mass and local pharyngeal disorders, including foreign body sensation, dysphagia, naso-obstruction and even intermittent bleeding (8).

The etiology and pathogenesis of FDCS are not clear. It is possible that certain FDCSs, particularly in hepatic and splenic lesions (10–12), are associated with EBV infection, although the association is not evident in pharyngeal tumors as the *in situ* hybridizations for EBV-encoded RNA were negative in the study by Duan *et al*(8), as well as the present study. Pauwels *et al*(13) and Chan *et al*(14) agreed that FDCS developed according to a hyperplasia-dysplasia-neoplasia sequence in follicular dendritic cells.

The diagnosis of pharyngeal FDCS depends on pathology. Histologically, the typical lesion characteristics are whorl, storiform and fascicular arrangements of oval to spindle tumor cells with indistinct cell borders, possible syncytial growth patterns and pale eosinophilic cytoplasm (5,15,16). Infiltrating small lymphocytes, which may gather around blood vessels, creating a cuffing pattern (5,16), may be observed in the background. The nuclei of the tumor cells may be elongated or oval with delicate dispersed chromatin and small nucleoli (5,16). The cells may also be multinucleated. Nuclear atypia and mitotic activity vary among different lesions (5,16).

Due to the previously mentioned morphological variability, immunohistochemical confirmation is essential. As demonstrated in the literature, the most sensitive and specific markers for FDCS are CD21, CD23 and CD35 (17). They are consistently positive and should be the first-line markers. D2-40 and CXCL13 were also considered to be effective by Xie *et al*(18) and Vermi *et al*(19) and should be observed together with other immunohistochemical makers including vimentin, S-100 protein, EMA and CD68, which were variably positive (17,20). CD1a, HMB45 and CD34 are specifically negative while CK is occasionally positive in FDCS (2,15,21). Among the three present cases, CD21, CD23, CD35 and D2-40 were all distinctly positive. CD3 and CD20 (17) have been noted to be negative in FDC tumor cells but positive in the background lymphocytes, but were replaced by LCA (leukocyte common antigen) (20) and CD163 in the present study. Moreover, EGFR was observed to be overexpressed and may be a therapeutic target (17). Clusterin was reported to be an important marker in the differential diagnosis of FDCS (22). Several other variable antibodies, including desmoplakin, fascin, bcl-2 and p53 and certain nonspecific markers, including smooth muscle actin (SMA), CD30, lysozyme and myeloperoxidase (MPO), are used clinically (5,7,17,20,21,23,24). The Ki-67 antigen expression levels were low to moderate (15). EBER staining by *in situ* hybridization was negative in all pharyngeal FDCS cases, according to the statistics of Duan *et al*(8). In the three present cases, the Ki-67 LIs were 35, 40 and 30%, respectively, and none of the cases were positive for EBER.

Misdiagnosis occurs frequently due to the rarity of pharyngeal FDCS tumors and similar histopathological characteristics shared with other common pharyngeal tumors, including ectopic meningioma, undifferentiated carcinoma, nerve sheath tumor, inflammatory sarcoma, squamous cell carcinoma, lymphoma and fibrous histiocytoma (2,5,9,14,20,25–32). All three present cases were misdiagnosed. The misdiagnosis rate in the present survey was 57% (26/46), higher than the 30% reported by Shia *et al*(31) in the whole body. Among the mistaken diagnoses, ectopic meningioma accounted for 12% (3/26) and undifferentiated carcinoma for 19% (5/26). Ectopic meningioma and FDCS feature syncytial cells arranged in whorl patterns and the tumor cells are negative for CK. However, lymphocytes and positive reactions for CD21, CD68 and CD35 are not observed in meningiomas (33). Similar to FDCS, undifferentiated carcinoma shows syncytial cells with vesicular nuclei and infiltrating lymphocytes. However, immunohistochemical markers positive for CD21, CD35 and CD23 and negative for CK, may be used to distinguish undifferentiated carcinoma from FDCS (8). Whenever pharyngeal tumors are identified, we recommend differential diagnosis and specific monoclonal antibody staining for FDCS.

FDCS is considered to be a sarcoma of intermediate-grade malignancy, which was demonstrated by Chan *et al*(5) in the observation of 17 cases. Prior to Chan *et al*’s study, it was regarded as an indolent tumor similar to a low-grade soft tissue sarcoma with a tendency for local recurrence and a low risk of metastasis (2,25). However, after analyzing 20 cases, Domínguez-Malagón *et al*(9) suggested that, in the pharyngeal region, FDCS is a low-grade carcinoma with recurrence, metastasis and mortality rates of 25, 25 and 5%, respectively. Duan *et al*(8) also suggested this and the rates of their 41-case analysis were 23, 21 and 3%, respectively. In the present study of 52 cases, the overall recurrence, metastasis and mortality rates were 40, 16 and 10%, respectively. The 2- and 5-year DFS rates were 66.2 and 51.3%, respectively. This demonstrated that extranodal FDCS of the pharyngeal region remains low-grade with recurrence tendencies.

FDCSs of various anatomical sites in the pharyngeal region were also analyzed. The most common site was the tonsils, where 52% of all cases occurred. The site with the poorest prognosis was the parapharyngeal space, with recurrence, metastasis and mortality rates of 80, 20 and 30%, respectively. The recurrence, metastasis and mortality rates of FDCS at five sites were calculated and survival curves were shown for the first time. However, larger-scale research is required due to the limited number of cases in certain sites, particularly the nasopharynx, palate and pharynx.

As a result of the rarity of FDCS, the assessment of pharyngeal FDCS prognoses remains difficult. Li *et al*(7) proposed a model for recurrence risk assessment according to the tumor size and histological grade. By this model, extranodal FDC sarcomas were divided into low-, intermediate- and high-risk groups and the recurrence rates were 16, 46 and 73%, respectively, while the mortality rates were 0, 4 and 45%. In the present study, statistical analyses suggested that large tumors (≥4 cm in the longitudinal dimension) had a worse outcome compared with small tumors (<4 cm).

There is not yet a consensus with regard to the optimal therapeutic modality due to the limited number of case reports and absence of prospective studies of treatments and outcomes. Surgery or combination therapy (surgery followed by chemotherapy and/or radiotherapy) are the choices for the initial treatment. Radical surgery (dissection of nodal lesions or wide local excision of extranodal lesions) is essential for localized FDC tumors, as demonstrated by De Pas *et al*(34). Chera *et al*(35) analyzed 67 cases of FDCS in the head and neck region and recommended postoperative radiotherapy. The total doses ranged between 6,000 and 7,000 cGy. With regard to chemotherapy, the agents most commonly used are combination regimens designed for the treatment of non-Hodgkin’s lymphoma, such as the CHOP (cyclophosphamide, doxorubicin, vincristine and prednisone) or CHOP-like regimens (36). Other regimens, such as sarcoma regimens, usually include a combination of doxorubicin and ifosfamide or gemcitabine, while a taxane may also be selected (17). Generally, 3 to 4 courses of chemotherapy were performed and partial or complete responses were achieved. However, it is not clear whether adjuvant treatment (chemotherapy and/or radiotherapy following surgery) is essential. Adjuvant treatment made no difference according to Duan *et al*(8) who analyzed 42 cases in the pharyngeal region. However, Karabulut *et al*(37) suggested that it was better to administer radiotherapy following resection to prevent local recurrence. According to the results of the present 52-case study, the average disease-free time of patients who underwent adjuvant treatment (range, 6 to 180 months; mean, 39 months) was longer compared with patients who underwent surgery alone (range, 2 to 120; mean, 24 months). The recurrence and mortality rates of the group who underwent surgery and adjuvant therapy were 46.2 and 7.7%, respectively, while those of the surgery alone group were 34.8 and 8.7%. However, the survival curves exhibited no statistically significant differences in DFS and OS rates between the two groups. From our clinical experience, postoperative adjuvant therapy appears to contribute to a longer disease-free time and is recommended. However, this remains to be clarified by larger-scale prospective studies of the treatments and outcomes of FDCS.

For accurate identification and effective therapy for FDCS, more attention should be paid to diagnosis and treatment.

## Figures and Tables

**Table I:** Clinical characteristics.

Case	Age (years)	Gender	Site	Symptom	Size (cm)	Initial diagnosis	Initial treatment	Recurrence, DFT (months)	Status	Follow-up (months)
1	36	F	Tonsil, CLN	Oropharyngeal mass, slight dysphagia, 1 month	3.0×2.5×1.5	Non-specific inflammation	Surgery	LR, 6	AWD	15
2	64	F	Parapharyngeal space	Oropharyngeal mass, dysphagia, 12 months	6.0×4.0×3.0	Squamous cell carcinoma	Surgery, ChT	NA	STD	7
3	59	F	Tonsil	Oropharyngeal mass, dysphagia, dyspnea, 2 months	4.5×4.0×2.0	Benign tumor	Surgery	LR, 17	STD	24

F, female; M, male; CLN, cervical lymph nodes; ChT, chemotherapy; RT, radiotherapy; LR, local recurrence; AWD, alive with disease; NED, no evidence of disease; STD, succumbed to disease;

NA, not available.

**Table II:** Immunohistochemical characteristics.

Case	CD21	CD35	CD23	D2 40	CXCL 13	S-100	Vimentin	EMA	CK	CD163	LCA	CD1a	CD68	CD34	CD3	CD20	Ki-67 (%)	EBER
1	+	+++	+++	+	−	−	+	−	−	+b	+b	0	0	0	0	0	35	−
2	++	+	+++	+	−	−	+	−	−	+b	+b	0	0	−	0	0	40	−
3	+	+	++	+	−	−	+	+	+	+b	+b	−	−	0	−	−	30	−

D2-40, podoplanin; EMA, epithelial membrane antigen; CK, cytokeratin; LCA, leukocyte common antigen; +, positive; −, negative; 0, not done; +b, positive for background cells, negative for tumor cells.

**Table III:** Clinical characteristics of 49 patients with pharyngeal FDC sarcoma from literature (case 4–52).

Case no.	Reference	Age (years)	Gender	Size (cm)	Sites	Initial diagnosis	Initial treatment	Recurrence, DFT (months)	Status	Follow-up (months)
4	Chan *et al*(38), 1994	44	M	1.5	Tonsil	FDC tumor	Surg	NA	NED	36
5	Chan *et al*(38), 1994	63	F	3×3×2	Soft palate	Acinic cell carcinoma	Surg + RT	NA	NED	54
6	Perez-Ordonez *et al*(39), 1996	62	F	NA	Tonsil	NA	Surg	NA	NED	12
7	Nayler *et al*(40), 1996	18	F	4×2×2	Tonsil	FDC tumor	Surg + ChT	NA	NA	NA
8	Chan *et al*(5), 1997	32	M	NA	Tonsil	NA	Surg + RT	LR & DM (LN), 54	AWD	54
9	Chan *et al*(5), 1997	40	F	7×3×2	Parapharyngeal space	NA	Surg	LR, 12	AWD	12
10	Beham-Schmid *et al*(41), 1998	44	M	2	Nasopharynx	FDC tumor	Surg + RT + ChT	NA	NED	20
11	Desai *et al*(20), 1999	45	F	6×3×3	Parapharyngeal space	Ectopic meningioma	Surg	LR, 31	NED	57
12	Araujo *et al*(25), 1999	14	M	1.5×1.0×0.5	Palate	Fibrous histiocytoma	Surg	NA	NED	5
13	Chan *et al*(14), 2001	23	M	1	Nasopharynx	No specific inflammation	Surg	NA	NED	36
14	Vargas *et al*(42), 2002	54	F	3	Tonsil, CLN	FDC tumor	Surg	NA	NED	8
15	Vargas *et al*(42), 2002	54	F	6	Parapharyngeal space, parotid	FDC tumor	Surg + RT	LR, 6	AWD	8
16	Biddle *et al*(2), 2002	48	M	3.5×2×2	Tonsil	FDC sarcoma	Surg	NA	NED	8
17	Biddle *et al*(2), 2002	48	F	1.5×1.2×1.0	Tonsil, CLN	Low-grade malignancy	Surg	NA	NED	6
18	Biddle *et al*(2), 2002	33	M	NA	Pharynx	Malignant schwannoma	Surg + RT	DM (lung), 10	AWD	10
19	Tish *et al*(36), 2003	51	M	NA	Tonsil	FDC sarcoma	Surg + RT	NA	NED	60
20	Satoh *et al*(26), 2003	16	M	3.0×2.5	Parapharyngeal space, palate	Low-grade malignancy	Surg + RT + ChT	NA	NED	24
21	Wang *et al*(27), 2003	59	M	3	Pharynx	NESCTWEF^*^	Surg	NA	NED	48
22	Dominguez-Malagon *et al*(9), 2004	29	F	4.8	Parapharyngeal space	Malignant meningioma	Surg + RT	LR, 12	STD	132
23	Dominguez-Malagon *et al*(9), 2004	48	M	1.5	Tonsil, CLN	FDC sarcoma	Surg + RT	NA	NED	36
24	Dominguez Malagon *et al*(9), 2004	26	M	NA	Parapharyngeal space	FDC sarcoma	Surg + RT + ChT	DM (lung), 36	AWD	72
25	Idrees *et al*(28), 2004	77	F	NA	Tonsil	SCC	Surg + RT	DM (LN, lung), 96	AWD	96
26	Georglass *et al*(43), 2004	61	M	5×4	Hypopharynx, CLN	FDC sarcoma	Surg	NA	NA	NA
27	Grogg *et al*(44), 2004	57	F	NA	Tonsil, CLN, ALN	NA	Surveillance	NA	AWD	8
28	Bothra *et al*(29), 2005	45	M	NA	Tonsil	UD-carcinoma	Surg	NA	NED	12
29	Bothra *et al*(29), 2005	45	M	NA	Tonsil	UD-carcinoma	Surg	NA	NED	12
30	Bothra *et al*(29), 2005	34	M	NA	Tonsil	FDC sarcoma	Surg	LR, 120	AWD	120
31	Chou *et al*(45), 2005	61	M	NA	Soft palate	NA	Surg	NA	NED	60
32	Aydin *et al*(33), 2006	76	F	3.5×3.5×1.5	Tonsil	FDC sarcoma	Surg + RT	NA	NED	48
33	Clement *et al*(30), 2006	27	F	4×3×2	Tonsil	Nerve sheath tumor	Surg + RT	NA	NED	6
34	Shia *et al*(31), 2006	69	F	NA	Tonsil	SCC	Surg + RT	DM (LN, lung), 96	AWD	108
35	McDuffie*et al*(46), 2007	59	F	4	Tonsil	FDC sarcoma	Surg + RT	NA	NED	18
36	Fan *et al*(32), 2007	48	F	NA	Tonsil	Malignant lymphoma	Surg + RT + ChT	LR&DM (LN), 180	AWD	180
37	Fan *et al*(32), 2007	42	M	NA	Nasopharynx	FDC sarcoma	Surg + RT + ChT	DM (LN), 48	AWD	144
38	Alexander *et al*(47), 2007	69	M	3	Parapharyngeal space	Paraganglioma	Surg	LR, 12	AWD	12
39	Soriano *et al*(17), 2007	33	M	NA	Nasopharynx	NA	Surg + RT	LR, 10	STD	14
40	Encabo *et al*(48), 2008	36	F	2.3×2.3	Nasopharynx	Teratocarcinosarcoma	Surg + RT	NA	NED	27
41	Vaideeswar *et al*(49), 2009	50	M	2×2	Tonsil	UD carcinoma	Surg	NA	NED	48
42	Duan *et al*(8), 2010	54	F	3×3×2	Nasopharynx	UD-carcinoma	Surg + RT	NA	NED	18
43	Duan *et al*(8), 2010	41	M	3×3×2	Nasopharynx	UD carcinoma	Surg	NA	NED	7
44	Duan *et al*(8), 2010	41	M	3×3×2	Tonsil	FDC sarcoma	Surg	NA	NED	9
45	Duan *et al*(8), 2010	39	F	2×2×1	Soft palate	Ectopic meningioma	Surg	NA	NED	40
46	Li *et al*(7), 2010	60	M	5	Tonsil	Granuloma	Surg + RT	NA	NED	86
47	Li *et al*(7), 2010	35	F	5	Parapharyngeal space	Nasopharyngeal sarcoma	Surg	LR, 2	STD	12
48	Li *et al*(7), 2010	28	F	6	Parapharyngeal space	FDC sarcoma	Surg + RT + ChT	DM (lung), 14	AWD	22
49	Li *et al*(7), 2010	55	M	2	Tonsil	FDC sarcoma	Surg + RT	LR, 18	AWD	21
50	Young *et al*(50), 2010	65	M	3×2×1.7	Tonsil	FDC sarcoma	Surg + RT	NA	NED	24
51	Suchitha *et al*(51), 2010	63	M	4.2×4×2	Tonsil	FDC sarcoma	Surg + RT	NA	NED	8
52	Karabulut *et al*(37), 2012	70	F	3×3×2	Nasopharynx	FDC sarcoma	Surg	LR, 12	AWD	46

F, female; M, male; CLN, cervical lymph nodes; ALN, axillary lymph nodes; surg, surgery; ChT, chemotherapy; RT, radiotherapy; AWD, alive with disease; NED, no evidence of disease; STD, succumbed to disease; NA, not available; LR, local recurrence; DM, distant metastasis; DFT, disease free time following surgery; LN, lymph node; FDC, follicular dendritic cell; NESCTWEF, nonepithelial spindle cell tumor with epithelial features; UD-carcinoma, undifferentiated carcinoma; SCC, squamous cell carcinoma.

**Table IV:** Survival rates.

		DFS rate (%)		OS rate (%)					P-value	
Cases	Frequency (n/total, %)	2-year	5-year	2-year	5-year	Recurrence rate (%)	Metastasis rate (%)	Mortality rate (%)	DFS	OS
Available total	50, 100	66.2	51.3	88.6	88.6	40	16	10	-	-
Surgery alone	23/50, 46	67.4	56.2	84.7	84.7	34.8	0	8.7	0.5513	0.7193
Surgery + RT/ChT	26/50, 52	73.8	66.4	91.4	91.4	46.2	30.8	7.7		
Size <4 cm	23/35, 66	81.5	-	100	-	17.4	0	0	0.0446	0.0086
Size ≤4 cm	12/35, 34	36.8	36.8	59.7	59.7	58.3	8.3	33.3		
Tonsil	27/52, 52	95.8	66.4	95.3	88.6	30.8	15.4	3.9	-	-
Parapharyngeal space	10/52, 19	40.0	40.0	95.3	88.6	80	20	30	-	-
Nasopharynx	8/52, 15	71.4	71.4	95.3	88.6	37.5	12.5	12.5	-	-
Palate	4/52, 8	100	100	95.3	88.6	0	0	0	-	-
Pharynx	2/52, 4	50.0	50.0	95.3	88.6	50	50	0	-	-

RT, radiotherapy; ChT, chemotherapy; -, not available; LR, local recurrence; DM, distant metastasis; DFS, disease free survival; OS, overall survival.
